# The Proton in Biochemistry: Impacts on Bioenergetics, Biophysical Chemistry, and Bioorganic Chemistry

**DOI:** 10.3389/fmolb.2021.764099

**Published:** 2021-11-26

**Authors:** Todd P. Silverstein

**Affiliations:** Chemistry Department (emeritus), Willamette University, Salem, OR, United States

**Keywords:** acidity, pH, aqueous solution, acid catalysis, enzyme catalysis, proton transfer, proton transport, diffusion

## Abstract

The proton is the smallest atomic particle, and in aqueous solution it is the smallest hydrated ion, having only two waters in its first hydration shell. In this article we survey key aspects of the proton in chemistry and biochemistry, starting with the definitions of pH and p*K*_a_ and their application inside biological cells. This includes an exploration of pH in nanoscale spaces, distinguishing between bulk and interfacial phases. We survey the Eigen and Zundel models of the structure of the hydrated proton, and how these can be used to explain: a) the behavior of protons at the water-hydrophobic interface, and b) the extraordinarily high mobility of protons in bulk water via Grotthuss hopping, and inside proteins via proton wires. Lastly, we survey key aspects of the effect of proton concentration and proton transfer on biochemical reactions including ligand binding and enzyme catalysis, as well as pH effects on biochemical thermodynamics, including the Chemiosmotic Theory. We find, for example, that the spontaneity of ATP hydrolysis at pH ≥ 7 is not due to any inherent property of ATP (or ADP or phosphate), but rather to the low concentration of H^+^. Additionally, we show that acidification due to fermentation does not derive from the organic acid waste products, but rather from the proton produced by ATP hydrolysis.

## 1 Introduction

For such a tiny particle, the proton^1^ certainly packs a wallop. It is so important that most laboratories in the world routinely measure its concentration (i.e., pH), and many compounds are judged by their ability to release it into solution. H^+^ can make many reactions go faster (acid-catalyzed) and/or further (pH-dependent reaction free energy). It diffuses faster in water than any other ion, and in fact, its diffusion in proteins and membranes is key to many biochemical processes.

In this review we will summarize current knowledge on the structure of H^+^_(aq)_, and its significance in biology and biochemistry. Starting with the definitions of pH, *K*_w_, and p*K*_a_, we explore how they are applied inside biological cells, in nanoscale spaces, and in bulk vs. interfacial phases. We then survey the Eigen and Zundel models of the hydrated proton, and apply these models to protons at interfaces, and proton mobility in water (via Grotthuss hopping) and in proteins (via proton wires). In our discussion of biochemical thermodynamics and kinetics we survey of the effects of pH on protein structure, ligand binding, and enzyme catalysis. We complete our discussion of biochemical thermodynamics by exploring the effects of pH on ATP hydrolysis, Chemiosmotic Theory, and fermentation.

### 1.1 H^+^ Concentration (pH) in Chemistry and Biology

Because H^+^ concentration is normally very low and can vary over several orders of magnitude, it is measured on a log-scale:

$$\text{pH}\equiv \;-\text{log}\left({\text{a}}_{\text{H}+}\right)\;=\;-\text{log}\left(\gamma_{\text{H}+}\text{·}\left[{\text{H}}^{+}\right]/\text{c°}\right)\;\approx \;-\text{log}\left[{\text{H}}^{+}\right]$$ (1)

where *a* = activity, *γ* = activity coefficient, and *c*° = 1 M, the standard state concentration. Because *γ* ≈ 1 for concentrations below 0.01 M, the approximation in Eq. 1, pH ≈ -log [H^+^], holds true above pH 2. (The Hammett acidity function (Hammett and Deyrup, 1932) and other acidity scales (Ivanov et al., 2021) have been developed for pH < 2.).

Pure water auto-ionizes to give H^+^ and OH^−^:

$${\text{H}}_{2}O_{\left(1\right)}⇌{\text{H}}_{\left(\text{aq}\right)}^{+}+OH_{\left(\text{aq}\right)}^{-}$$ (2)

$$K_{w}=\;\frac{a_{H^{+}}\cdot a_{OH^{-}}}{a_{H_{2}O}}=\;a_{H+}\cdot a_{OH-}\approx \left[H^{+}\right]\left[OH^{-}\right]$$ (3)

Because the equilibrium constant for this reaction at 25°C is K_w_ = 1.00 × 10^−14^, the pH of pure water is 7 ([H^+^] = 1.00 × 10^−7^ M), and this constitutes a neutral solution. A pH close to neutrality is maintained in the cytoplasm, as well as in several other spaces both inside and outside the cell (Table 1). Because the main products of cellular respiration are organic acids (anaerobic fermentation) and CO_2_ (aerobic), and hydrated CO_2_ is mostly carbonic acid (H_2_CO_3_), maintaining a neutral cytoplasmic pH (pH_cyt_) requires active proton efflux, maintained by various H^+^ pumps and channels, as we will discuss below. These “waste” protons can be pumped into the extracellular space (especially for acid-secreting cells like those lining the stomach), or into various acidic intracellular compartments (e.g., endosomes, lysosomes; see Table 1). Furthermore, proton pumping across bioenergetic membranes is a key component of chemiosmotic ATP synthesis in mitochondria, chloroplasts, and bacteria.

**TABLE 1:** pH of cellular compartments under normal (non-disease state) conditions.

Compartment	pH^a^	Compartment	pH^a^
Extracellular space	7.4	Early endosomes	5.8–6.4
Cytoplasm	7.1–7.3	Late endosomes	5.4–5.8
Nucleus	7.2–7.3	Secretory granules	5.5
Endoplasmic reticulum	7.3	Lysosomes	4.5–5.0
Cis Golgi lumen	6.7	Mitochondrial matrix	7.3–8.1^b^
Trans Golgi lumen	6.0	Mitochondrial intermembrane space	6.8–7.2^b^
Peroxisomes	7.0	Chloroplast stroma	8
		Thylakoid disk lumen	5.5–6

a Measured pH values from (Casey et al., 2010) and (Jaworska et al., 2021).

b pH measurements in mitochondria have improved over the years, correcting for various systematic errors. Recent measurements have clustered around 7.3–7.6 in the matrix, and 7.1–7.2 in the intermembrane space.

Maintaining the proper pH is important to optimize many biochemical processes (Pouyssegur et al., 1985), especially enzyme catalysis, as we shall discuss below. It is thus not surprising that there are a number of disease states that are characterized by excessively low pH (acidosis/acidemia) or high pH (alkalosis/alkalemia). For example, the acidosis common in Alzheimers Diseased neurons (possibly caused by ischemic low oxygen and excessive anaerobic fermentation) is believed to increase production of the toxic Aβ peptide and Ca^2+^ influx, triggering cell death by apoptosis (Fang et al., 2010). Meanwhile, cancer cells are also ischemic, so in order to avoid acid-induced apoptosis, they over-pump excess protons out of the cell (Fang et al., 2010). This allows tumor cells to maintain a slightly alkaline interior (pH_cyt_ 7.4) and an acidified exterior (pH_ext_ = 6.5–6.8). These pH changes have been shown to account for the increased cell proliferation in a tumor, as well as the cytoskeletal and extracellular matrix remodeling, cell migration, and tissue invasion that are characteristic of metastasis (Damaghi et al., 2013). Cancer cells maintain such a tight regulation of their disease state pH values (Korenchan and Flavell, 2019) (Quach et al., 2016) (Swietach, 2019) that pH readjustment has been proposed as a possible anti-cancer therapy (Korenchan and Flavell, 2019). The relationship between pH regulation and anaerobic metabolism in cancer and Alzheimers Disease was recently reviewed (Schwartz et al., 2020).

### 1.2 Does Size Matter? Protons in Nanoscale Spaces

It has been known for quite some time that some sub-cellular compartments are so small that, based on the measured pH, only a few free protons (or perhaps even less than one!) should exist inside the compartment. For example, mitochondria typically occupy about 1 fL, of which 90% is matrix and 10% intermembrane space (Bal et al., 2012). The matrix at a pH of 8 should thus contain only 5.4 free protons; similarly, the intermembrane space (pH 7) will only have 6 free protons. Thylakoid disks contain even fewer protons: They comprise 0.3 to 1.9 aL, so at a typical pH of 5.6, the largest ones will have 2.9 free protons in their lumen, and the smallest only 0.5 (Bal et al., 2012).

This raises three important questions: First, what is the meaning of a fraction of a solute ion? This question has two answers. For solutes that are unbuffered (e.g., nucleotides, metabolites), each individual compartment will contain a range of integer numbers of solute molecules. For example, 650 nm radius compartments containing a solution of 8.4 nM DNA should contain, on average, 1.3 solute molecules. Shon and Cohen found a range of solute per compartment integer numbers ranging from 0 to 5; the probability histogram for each compartment integral number, showing the stochastic nature of the distribution, fit nicely to a Poisson distribution.(Shon and Cohen, 2012).

For solutes that are buffered (e.g., H^+^, Ca^2+^, and Mg^2+^), Bal et al. pointed out that due to the small size of nanoscale compartments, the concentration of supposedly “free” ions must be reported by soluble probes. These probes actually report their binding equilibrium state; from calibration curves obtained in macroscale solutions (i.e., test tubes), the nanoscale equilibrium is converted to a concentration of “free” ion. However, in buffered nanoscale compartments, the probe could well be in equilibrium with buffers rather than free ions.

For example, consider the smallest thylakoid disk, with its pH of 5.6 corresponding to only 0.5 free protons. In addition to the stochastic Poisson distribution explanation above (a population of disks that contain 0, 1, or 2 protons, averaging 0.5), if the disks contain mM concentrations of phosphates (e.g., H_2_PO_4_^−^, HATP^3−^, HADP^2−^) with p*K*_a_ ≈ 6.5, then there need be no free protons at all in order for the probe to report a pH of 5.6. Assuming that the p*K*_a_ of the reporter probe is 6.0, we have acid ionization equilibria for both the probe (HX) and phosphate (H_2_P_i_^−^):

$$\text{HX}\to {\text{H}}^{+}{+\text{ X}}^{-}{\text{K}}_{\text{a,X}}\;=\;{\left[{\text{H}}^{+}\right]}_{\text{eq}}{\left[{\text{X}}^{-}\right]}_{\text{eq}}/{\left[\text{HX}\right]}_{\text{eq}}{=10}^{-6\text{.0}}$$ (4)

$${\text{H}}_{2}{\text{P}}_{\text{i}}^{-}\to {\text{H}}^{+}{+\text{ HP}}_{\text{i}}^{2-}{\text{K}}_{\text{a,P}}\;=\;{\left[{\text{H}}^{+}\right]}_{\text{eq}}{\left[{\text{HP}}_{\text{i}}^{2-}\right]}_{\text{eq}}/{\left[{\text{H}}_{2}{\text{P}}_{\text{i}}^{-}\right]}_{\text{eq}}{=10}^{-6\text{.}5}$$ (5)

At pH 5.6, we can calculate that the ratio [HX]_eq_/[X^−^]_eq_ = 2.5. Thus a probe [HX]/[X^−^] ratio of 2.5 in the smallest thylakoid disk would be interpreted as 0.5 free protons per disk. However, we can also consider the probe/phosphate equilibrium:

$$\begin{array}{l} {\text{X}}^{-}{+\text{ H}}_{2}{\text{P}}_{i}^{-}\to {\text{HX}+\text{ HP}}_{i}^{2-}\\ {\text{K}}_{\text{eq}}\;=\;\frac{{\left[HX\right]}_{eq}{\left[HP_{i}^{2-}\right]}_{eq}}{{\left[X^{-}\right]}_{eq}{\left[H_{2}P_{i}^{-}\right]}_{eq}}=\;\frac{10^{-6.5}}{10^{-6.0}}=0.316 \end{array}$$ (6)

From Eq. 6 we know that as long as the phosphate [H_2_P_i_^−^]_eq_/[HP_i_^2−^]_eq_ ratio is 7.94, then the probe [HX]/[X^−^] ratio at equilibrium will be 2.5, and this would be interpreted as a pH of 5.6, even in the absence of *any* free protons inside the thylakoid disk.

The second, related question is: How can bioenergetic proton pumping and proton flow-driven ATP synthesis occur at biological rates if so few free protons exist in these bioenergetic compartments? For example, how can an F_1_F_0_ ATP synthase molecule in a chloroplast thylakoid disk membrane carry out H^+^-driven ATP synthesis if less than one free proton is present in the disk lumen? There are two significantly different and equally important answers to this question. First, the F_1_F_0_ ATP synthase is a rotary engine; protons flow spontaneously through the F_0_ channel, and the free energy is used to “spring-load” a rotary twist of a ratchet that stores mechanical energy (Stewart et al., 2014) (Junge and Nelson, 2015) (Kühlbrandt and Davies, 2016). Once a specific number of protons^2^ flow through the mammalian mitochondrial F_0_, mechanical energy in the ratchet is sufficient to release 3 newly synthesized ATP molecules from the F_1_ portion of the enzyme (Silverstein, 2014). A key mechanistic point is that protons flow through the F_0_ portion one at a time, and bioenergetic bulk phases contain copious amounts of weak acids (e.g., phosphates, carboxylates, amines) (Poznanski et al., 2013), as do protein side chains at the surface of bioenergetic membranes (Bal et al., 2012). As long as one of these groups ionizes to release a proton each time one disappears from the bulk phase, the pH in the bulk will remain constant; ATP can be synthesized and the engine will continue to run.

We must also consider a possible difference between the bulk phase, where pH is measured, and the membrane/bulk interface, i.e., the membrane surface. The chemiosmotic theory as presented by Nobel Laureate Peter Mitchell (Mitchell, 1961) (Mitchell, 1966) posits the driving force for proton flow between the bulk phases on either side of the bioenergetic membrane. However, Mitchell’s compatriot RJP Williams placed the driving force along the membrane surface and within the membrane (Williams, 1961) (Williams, 1978). Much early evidence favored the importance of Mitchell’s delocalized bulk-to-bulk protonmotive force; however, by the late 1970s, substantial evidence supporting the importance of Williams’s localized surface-to-surface force was beginning to accumulate (Kell, 1979) (Ferguson, 1985), and the localized protonmotive force is now widely accepted (Dilley, 2004) (Mulkidjanian et al., 2006) (Brändén et al., 2006) (Lee J. W., 2020) (Lee JW., 2020) (Springer et al., 2011) (Weichselbaum et al., 2017) (Gutman and Nachliel, 1995) (Kotlyar et al., 1994) (Heberle et al., 1994) (Nachliel and Gutman, 1996) (Gabriel and Teissie, 1996) (Gopta et al., 1999) (Cherepanov et al., 2003) (Mulkidjanian et al., 2005) (Rieger et al., 2014) (Toth et al., 2020) (Nilsson et al., 2016) (Sjöholm et al., 2017) (Morelli et al., 2020). Therefore, protons driving ATP synthesis need not come from the bulk phase, and thus the number of free protons calculated from bulk phase pH measurements may be irrelevant to bioenergetic coupling, at least under some conditions (Ferguson, 1985) (Dilley, 2004). As long as enough protons are found near the membrane surface, proton pumping and ATP synthesis can proceed apace (Toth et al., 2020) (Mulkidjanian et al., 2006) (Lee J. W., 2020) (Gutman and Nachliel, 1995) (Cherepanov et al., 2003).

This difference between bulk and membrane surface protons raises a third question, namely, do the dynamics of water and protons in tiny spaces differ from those in bulk solution? This is an area of active study; Bal et al. have found, for example, that soluble pH probes behave differently in dilute macroscopic solution and inside cells and organelles (Żurawik et al., 2016), and this difference must be taken into account in order to obtain accurate values of intracellular pH. Similarly, Crans, and Levinger have used NMR to study pH dependent vanadate speciation in reverse micelles of different sizes (Crans and Levinger, 2012). Others have stressed that concentrations in nanoscale spaces that contain only a few molecules vary stochastically, averaging to that found in bulk solution (Shon and Cohen, 2012) (Goch and Bal, 2020).^3^

### 1.3 Acid Strength (pK_a_) in Chemistry and Biology

The tendency of an aqueous acid HA to release H^+^ into water is described by the acid ionization equilibrium:

$$HA⇌{\text{H}}_{\left(\text{aq}\right)}^{+}{+\text{A}}_{\left(\text{aq}\right)}^{-}$$ (7)

and its equilibrium constant:

$$K_{a}=\;\frac{a_{H+}a_{A-}}{a_{HA}}\approx \;\frac{{\left[H^{+}\right]}_{eq}{\left[A^{-}\right]}_{eq}}{{\left[HA\right]}_{eq}}$$ (8)

In the approximation above we assume that all concentrations are below 0.01 M, so all activity coefficients ≈1. Because *K*_a_ values are often quite low and range over several orders of magnitude, they are usually expressed on a log-scale, as p*K*_a_. Eq. 8 is often written as the Henderson-Hasselbalch equation:

$${\text{pH }=\text{ pK}}_{\text{a}}+\text{ log}\left(\frac{{\left[A^{-}\right]}_{eq}}{{\left[HA\right]}_{eq}}\right)$$ (9)

Like any equilibrium constant, *K*_a_ may change with temperature (if ∆*H*°_a_ ≠ 0) and with solvent (Heller and Silverstein, 2020).^4^ For example, the less polar a solvent is, the less stable are the charged products of HA ionization, hence the less spontaneous the reaction is (lower *K*_a_/higher p*K*_a_). This can also apply to weakly acidic side chains in a protein; for example, a hydrophobic local environment will lower the *K*_a_ (raise p*K*_a_) of neutral weak acids (asp/glu-COOH, cys-SH, tyr-OH; see Table 2) making them less acidic, while raising the *K*_a_ (lower p*K*_a_) of cationic weak acids (hisNH^+^, lys-NH_3_^+^; see Table 2) making them more acidic. A nearby positive side chain destabilizes the protonated form of the weak acid and stabilizes the conjugate base form, which makes the acid more acidic, raising its *K*_a_ (lower p*K*_a_); a nearby negative side chain does the opposite. As we see in Table 2, these effects can alter the p*K*_a_ of a protein side chain by up to ±5 units from its nominal value in water.

**TABLE 2:** Aqueous pK_a_ values for amino acids that can have significant concentrations of both protonated and deprotonated forms around pH 7. Data are from 541 pK_a_ values reported under various conditions for 78 folded proteins (Pace et al., 2009).

Amino acid	p*K*_a_ range (low—high)	Average p*K*_a_	Nominal^a^ p*K*_a_
Asp-COOH	0.5–9.2	3.5 ± 1.2	3.9
Glu-COOH	2.1–8.8	4.2 ± 0.9	4.3
His-imid ≡ NH^+^	2.4–9.2	6.6 ± 1.0	6.5
Cys-SH	2.5–11.1	6.8 ± 2.7	8.6
Tyr-OH	6.1–12.1	10.3 ± 1.2	9.8
Lys-NH_3_^+^	5.7–12.1	10.5 ± 1.1	10.4
C-terminus-COOH	2.4–5.9	3.3 ± 0.8	3.7
N-terminus-NH_3_^+^	6.8–9.1	7.7 ± 0.5	8.0

a Measured for the amino acid inserted in an alanine pentapeptide.

Proteins have six types of amino acid side chains that can change protonation state fairly easily during their activity cycle. Table 2, adapted from (Pace et al., 2009), shows that only histidine and the N-terminus have nominal p*K*_a_ values that would allow them to have significant concentrations of both protonated and deprotonated forms around pH 7. However, as we discussed above, the electrostatics of the local protein environment can dramatically alter the p*K*_a_ value of an amino acid side chain, as noted by the p*K*_a_ ranges tabulated in Table 2. Thus, aspartate and glutamate can have p*K*_a_ values as high as 9, while lysine and tyrosine can have values as low as 6. Interestingly, the average within-protein p*K*_a_ is essentially the same as its nominal p*K*_a_ for all amino acids but one. Cysteine seems often to be situated near a positive (or δ+) charge, thus lowering its p*K*_a_ by about two units, into the neutral range. This suggests that a deprotonated thiolate anion may be functionally significant for many proteins (e.g, cysteine proteases (Chapman et al., 1997) and protein tyrosine phosphatases (Kolmodin and Aqvist, 2001)).

The change in charge resulting from deprotonation, from 0 to −1 or +1 to 0, can trigger a number of important changes in protein structure and function (Schönichen et al., 2013). These changes allow some proteins to serve as *in vivo* sensors and regulators of pH (Schönichen et al., 2013) (Casey et al., 2010), and they also figure into optimizing enzyme activity both in terms of substrate binding and catalysis, as we shall discuss below.

It is worth noting briefly that the water auto-ionization equilibrium (Eq. 2) releases H^+^ into solution, hence we can say that p*K*_w_ = p*K*_a_ (water) = 14.00. Many organic chemistry textbooks and papers mistakenly use 55.3 M for the concentration of conjugate acid (HA = H_2_O) in Eq. 8, to obtain a value of p*K*_a_ (water) = 15.74. However, because water is the solvent, its activity is 1, and using its molar concentration in an equilibrium constant expression is thermodynamically incorrect (Meister et al., 2014) (Silverstein and Heller, 2017). Similarly, the release of aqueous H^+^ into water is sometimes written as:

$${\text{H}}_{3}{\text{O}}_{\left(\text{aq}\right)}^{+}{+\text{ H}}_{2}{\text{O}}_{\left(1\right)}⇌{\text{ H}}_{2}{\text{O}}_{\left(1\right)}{+\text{H}}_{3}{\text{O}}_{\left(\text{aq}\right)}^{+}$$ (10)

As is often the case, aqueous H^+^ is written as the hydronium ion (H_3_O^+^) in Eq. 10, but as we shall discuss below, this is *not* meant to signify the actual structure of H^+^_(aq)_. In any case, the equilibrium constant for the reaction in Eq. 10 must be 1 because products and reactants are identical, so p*K*_a_ (H^+^_(aq)_) = 0. We often see this value as −1.74 in the organic chemistry literature, but again, this value is incorrect, due to the mistaken use of the molar concentration of water, 55.3 M, rather than its activity, 1 (Meister et al., 2014) (Silverstein and Heller, 2017).

The p*K*_a_ of an acid tells us the acid strength of its protonated form as well as the base strength of its deprotonated form (low p*K*_a_ = strong acid, high p*K*_a_ = strong conjugate base). Thus, we can predict the spontaneity of the acid-base reaction between HA and B if we know the p*K*_a_ values of the two conjugate acids, HA and HB^+^:

$${\text{HA}}_{\left(\text{aq}\right)}{\;+\text{ B}}_{\left(\text{aq}\right)}⇌{\text{ HB}}_{\left(\text{aq}\right)}^{+}{\;+\text{ A}}_{\left(\text{aq}\right)}^{-}$$ (11)

$$K_{eq}=\;\;\frac{{\left[HB^{+}\right]}_{eq}{\left[A^{-}\right]}_{eq}}{{\left[HA\right]}_{eq}{\left[B\right]}_{eq}}=\;\text{Ka}\left(\text{HA}\right)/\text{Ka}\left({\text{HB}}^{+}\right)$$ (12)

So log (K_eq_) = pK_a_ (HB^+^) - pK_a_ (HA).

For a spontaneous reaction under standard conditions, *K*_eq_ > 1, so log (*K*_eq_) > 0; this in turn is true if p*K*_a_ (HB^+^) > p*K*_a_ (HA). Thus, if we define for any acid-base reaction ∆p*K*_a_ ≡ p*K*_a_ (HB^+^) — p*K*_a_ (HA), then the reaction will be spontaneous if ∆p*K*_a_ is positive.^5^

With this in mind, it is quite surprising that acid-base reactions featured in enzyme catalysis can be exceedingly nonspontaneous, with some ∆p*K*_a_ values ranging from −8 to −23 (Silverstein, 2021). The question of how such incredibly nonspontaneous reactions can be catalytically useful will be discussed below.

We close the Introduction by repeating the observation of Colin Wraight (Wraight, 2006) that the biological significance of protons shows up in two types of reactions: 1) proton transport, which can occur over long distances, in several steps, and often features a pre-organized path; and 2) chemical conversion or catalysis, in which the proton is transferred from a donor to an acceptor in a single step. We will further expand on these two types of reactions in our discussion below.

## 2 Discussion 1: Proton Transfer in Water, Proteins, and Organelles

### 2.1 Structure of the Hydrated Proton: Eigen vs. Zundel

**FIGURE 1:**
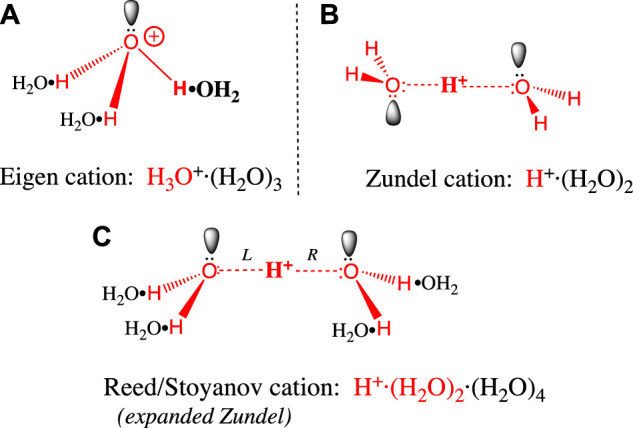
**(A)** The Eigen cation: H^+^, the large red bolded H on the right is covalently bonded to a single water in its first hydration shell. The three hydrogens in the resulting H_3_O^+^ (red) each form hydrogen bonds with a black water (H_2_O•) forming the second hydration shell. Bonds are not drawn to scale: O-H covalent bonds (red) have 0.96 Å bond lengths, whereas the H_2_O•H hydrogen bonds above are 1.4–1.7 Å long (Asthagiri et al., 2005) (Markovitch et al., 2008). **(B)** The Zundel cation: The central large red bolded H^+^ is shared equally between two inner shell waters (red), to form an H_5_O_2_^+^ dihydrate that features two symmetrical, unusually short and strong H-bonds. The symmetric trans conformation drawn here is the ground state in bulk water. **(C)** The Reed/Stoyanov cation: The Zundel dihydrate is expanded to include four black waters (H_2_O•) in a second hydration shell; the two central H-bonds in the dihydrate core are slightly longer and weaker than in the Zundel dihydrate (Reed, 2013). The asymmetric cis “sawhorse” conformation drawn here is believed to be the ground state at the hydrophobic phase/water interface.

Before we examine proton transfer reactions, it will help to understand the structure of the hydrated proton. As mentioned above, the (erroneous) assumption that the proton is hydrated by a single water molecule to give a hydronium ion (H_3_O^+^) is quite widespread in the literature. The two most widely accepted H^+^(aq) structures were posed in the 1960s by Eigen and Zundel (Eigen, 1964a) (Zundel, 1969), and both structures feature more than a single water of hydration. The Eigen cation (Figure 1A) is an H_9_O_4_^+^ tetrahydrate in which an H_3_O^+^ entity is hydrogen-bonded to three waters in a second hydration shell. These H-bonds are the “normal” type seen in bulk water and ice. The Zundel cation (Figure 1B) is an H_5_O_2_^+^ dihydrate where H^+^ is shared equally between two inner shell water molecules. The two central hydrogen bonds are symmetrical, and they are unusually short and strong (Beli et al., 1975). Stoyanov and Reed (Reed, 2013) have proposed a hexahydrate H_13_O_6_^+^ ion (Figure 1C) with a Zundel-like inner core (but with longer, symmetrical H-bonds) and four H-bonded water molecules in the second hydration shell. The proton’s +1 charge is delocalized over this entire hexahydrate complex (Reed, 2013).

Let us now evaluate the evidence supporting each of these structures. The Eigen tetrahydrate structure was hypothesized based solely on the greater mobility of H^+^_(aq)_ compared to hexahydrate metal ions in water (Eigen, 1964b) (Eigen, 1964a). The Zundel dihydrate structure was supported by the continuously broad absorbance features in the crude IR spectrum of H^+^_(aq)_ (Zundel, 1969). The Reed/Stoyanov structure was based on a precise measure of the hydration stoichiometry (i.e., six) and a detailed interpretation of its well-defined IR spectrum, obtained for the first time in 2010 (Stoyanov et al., 2010) (Stoyanov et al., 2011). This hexahydrate structure has recently been supported by several other studies (Thämer et al., 2015) (Daly et al., 2017) (Dahms et al., 2017) (Fournier et al., 2018) (Asthagiri et al., 2005); at this point it must be considered the definitive, most accepted structure (Reed, 2021).

Having said this, any static structural representation of the aqueous proton belies the extremely rapid kinetics of proton movement in water, a feature that is revealed experimentally by the very low activation energy for proton transfer (2.7 kcal/mol, (Cukierman, 2006)), and by the continuous broad absorption across its entire IR spectrum (Stoyanov et al., 2011). The presence of this broad IR absorption indicates that some H^+^_(aq)_ ions move more rapidly than the IR timescale, i.e., faster than normal vibrations. That the barriers to proton movement are so low gives sense to the idea that the Eigen and Zundel structures represent two idealized extremes (Botti et al., 2006) (Daly et al., 2017) that can easily interconvert: As one distorts the Zundel cation (Figure 1B) by shrinking the “L” H-bond toward 1 Å (i.e., covalent) and stretching the “R” H-bond toward 1.5 Å, one approaches the Eigen structure (Figure 1A). So, a distorted Zundel cation and a distorted Eigen cation could well represent the very same structure. In modeling the process of proton mobility, Agmon has stressed the importance of such Eigen/Zundel transitions (Markovitch et al., 2008). Indeed, many *in silico* studies arrive at descriptions of H^+^_(aq)_ having various admixtures of Eigen- and Zundel-like structures (Tuckerman et al., 1995) (Marx et al., 1999) (Botti et al., 2006) (Markovitch and Agmon, 2007) (Springer et al., 2011) (Asthagiri et al., 2005) (Biswas et al., 2017).

In summary, written as H_3_O^+^, the hydronium ion is a useful fiction but where feasible, should be replaced with the simple notation H^+^_(aq)_ as a signifier of the hexahydrate complex (Silverstein, 2014).

### 2.2 Protons at the Interface

Water’s two hydrogens can each donate an H-bond to a neighboring water molecule, while the two lone pairs on its oxygen can each accept an H-bond. Accordingly, liquid water forms a (mostly) tetrahedral H-bond network, as the average water molecule makes a total of 3.5–3.8 H-bonds (Luck, 1980) (Guardia et al., 2015) (Jorgensen and Madura, 1985) (AuthorAnonymous, 2021). On the contrary, in Figures 1A,C we see that the inner shell (red) water(s) bound to H^+^ make only 3 H-bonds. This is because the proton polarizes its inner shell waters in such a way as to make the remaining oxygen lone pair a very poor H-bond acceptor (Marx et al., 1999) (Markovitch and Agmon, 2007), which makes the hydrated proton a somewhat amphiphilic molecule; the 3 or 4 inner shell hydrogens at the bottom of the complex are quite polar, and the oxygen(s) at the top much less polar (Figures 1A,C). This in turn explains why molecular dynamics simulations have consistently found H^+^_(aq)_ to be enriched near the air-water (Vácha et al., 2007) (Lee and Tuckerman, 2009) (Tse et al., 2015) and decane-water interface (Zhang et al., 2012): The inner shell oxygen(s) point(s) toward the hydrophobic phase, while the hydrogens point back into the water phase, which is polar. By positioning the H+ complex thusly, hydrophobic repulsive forces are minimized, as is disruption of the H-bond network in the bulk water phase (Vácha et al., 2007) (Tse et al., 2015). This has implications for the retention of protons near the surface of biological membranes, and the delayed equilibration between surface and bulk protons, as envisioned by Williams’s localized surface-to-surface protonmotive force.

Evidence supporting the localized protonmotive force was cited in the Introduction (*Does Size Matter? Protons in Nanoscale Spaces*). In general, these experiments show that in many membrane systems, protons flow much faster along the membrane surface than they do between surface and bulk. In other words, there is an energy barrier than impedes surface to bulk proton equilibration. Three models have been proposed to explain this barrier. Proton binding to fixed charges at the membrane surface (e.g., lipid head groups) has been disproven by the weak dependence on pH (Mulkidjanian et al., 2005) and on lipid head group charge (Springer et al., 2011) (Sjöholm et al., 2017). Lee’s membrane capacitance model (Lee JW., 2020) requires a transmembrane electrochemical potential (negative inside) and posits disobedience of the Second Law of Thermodynamics (Lee J. W., 2020). However, as discussed above, the surface to bulk energy barrier is observed both at the air-water and decane-water interface, i.e., in the absence of any transmembrane electrochemical potential (i.e., ∆*µ*_H+_ = 0). This seems to rule out Lee’s model.

A promising model has been proposed by Junge and Mulkidjanian (Cherepanov et al., 2003) (Mulkidjanian et al., 2006) and supported by Pohl’s group (Zhang et al., 2012). This model considers the special features of the water layers adjacent to the membrane surface, whose dielectric constant of ≈10–30 is much lower than that of bulk water (80). The Born desolvation energy for a proton in this low dielectric surface water phase, along with surface electrostatic repulsion, provide a surface to bulk energy barrier of 4–11 kcal/mol (Cherepanov et al., 2003) (Zhang et al., 2012). This physical model explains the observations above that protons are enriched at the air-water interface where the amphiphilic hydrated proton is better accommodated in terms of hydrophobic force and water-water H-bond network disruption.

### 2.3 Proton Transfer and Mobility in Water

**FIGURE 2:**
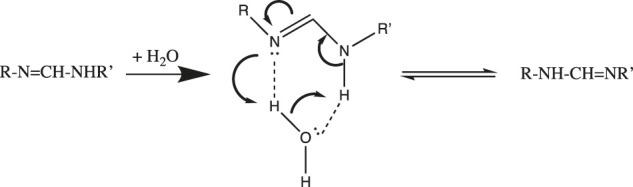
Water-catalyzed 1,3-tautomerization.

Now that we understand the structure of the hydrated proton, we can examine how it is transported. A simple example of the importance of water in proton transfer is seen in its catalysis of a 1,3-tautomerization (Maréchal, 2004). In Figure 2 we see that a single water molecule completes a stable 6-member ring with the RN=C-NHR′ substrate. The H-bond accepting and donating ability of this water allows nucleophilic attack by =N: to initiate a series of proton transfers that end with the RNH-CH=NR’ 1,3-tautomer.

**FIGURE 3:**
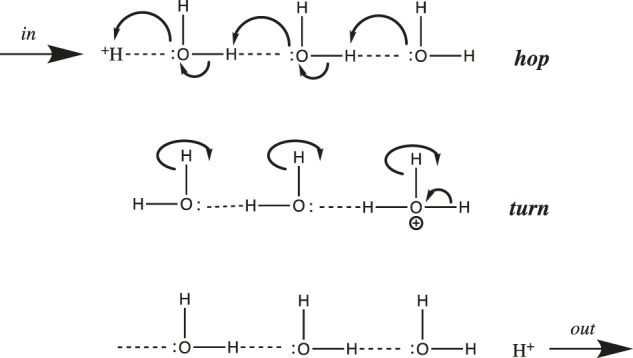
De Grotthuss proton hopping mechanism, with a chain of three waters, proton input from the left, and output to the right.

In general, proton transfer requires more than a single water molecule. From conductivity measurements made over two centuries ago, we know that the aqueous proton is far more mobile than any other cation. De Grotthuss hypothesized back in 1806 that in addition to translating “vehicularly” as a complete hydrated complex like other cations, H^+^ could also translate along a “bucket brigade” chain of water molecules (von Grotthuß, 1805) (De Grotthuss, 2006) (Cukierman, 2006). This speculative model, now known as the de Grotthuss proton “hopping” mechanism, turned out to be fairly accurate and quite useful. It is depicted in Figure 3 above for a chain of three water molecules:

De Grotthuss envisioned a two-step mechanism that begins with a pre-existing chain of hydrogen-bonded water molecules. After proton input (from the left in Figure 3), a sequence of oxygen nucleophilic attacks allows the proton to “hop” to the end of the chain. Then, in order to reset the chain in preparation for the next proton input, the waters must rotate or “turn back”, as the free proton exits (to the right in Figure 3).

Before we continue, a few provisos are in order regarding the scheme depicted in Figure 3. First, the choice of three waters is somewhat arbitrary; although entropy and Brownian motion put the upper limit at five molecules (Cukierman, 2006), molecular dynamics simulations (Markovitch and Agmon, 2007) (Knight and Voth, 2012) and IR spectroscopic results (Reed, 2013) suggest that the chain generally comprises two or three water molecules. Next, the linear one-dimensional chain is an oversimplification: water is bent (with a tetrahedral geometry including its two lone pairs), hence the chain must zig-zag in three dimensions. Thirdly, an assumption implicit in Figure 3 is that only a single water chain exists: All protons must enter at the same point on the left, and exit at the same point on the right. Using this assumption, it was shown that above 20°C, the rotation step was rate-determining, due to the number of H-bonds that had to be broken (Cukierman, 2006). However, for proton mobility in both bulk solution and at an interface, the rate-determining step has been shown to be hopping, not turning (Cukierman, 2006). This is true because in bulk solution and at a planar interface there are a myriad of different proton input points, and for each input point, many different paths and chains that the proton could follow. Hence, there is no requirement to reset the specific chosen path before the next proton can be input. Having said that, the single-chain assumption (with some modifications) is a reasonable approximation of what happens in proton channels and pores inside proteins; such chains were dubbed proton “wires” by Nagle and Morowitz (Nagle and Morowitz, 1978).

### 2.4 Proton Transfer and Mobility in Proteins

**FIGURE 4:**
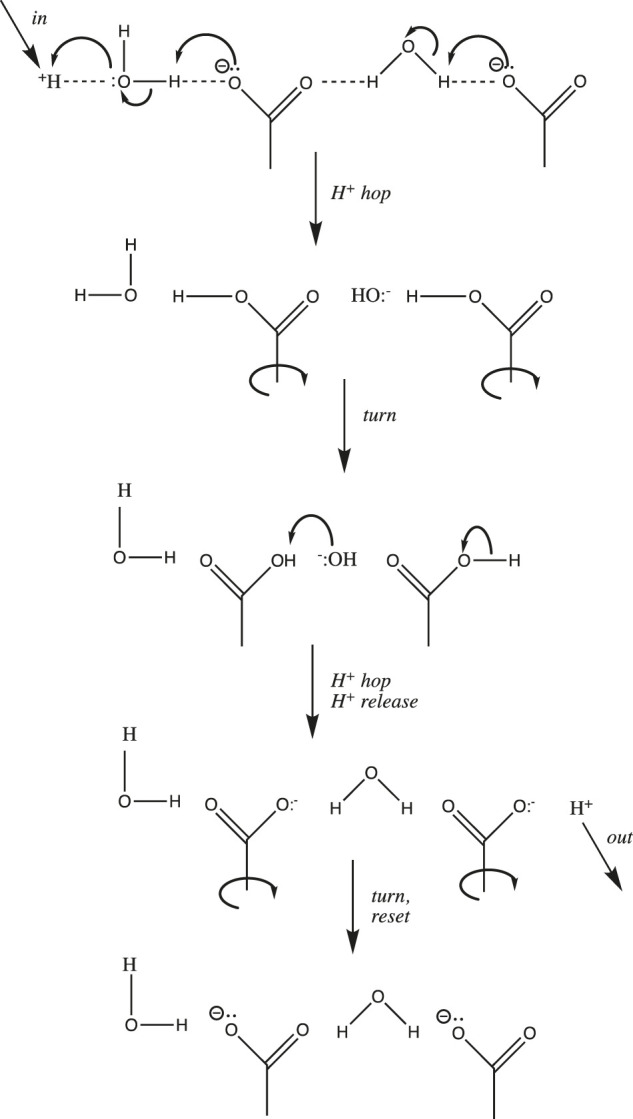
Simple proton wire comprising two water molecules and two carboxylate side chains.

Examples of proton wires have been found in both soluble and transmembrane proteins. Soluble enzymes that catalyze reactions that either consume or produce protons often have one or more proton wires leading from the external surface of the protein into its buried active site; these wires allow the facile shuttling of protons between the active site and the external solution. Transmembrane proton pumps and sinks have pores lined by proton wires that take up protons from one side of the membrane, shuttle them through the low dielectric interior of the membrane, and release them into the bulk solution on the opposite side. A protein-situated proton wire is a modified Grotthuss chain that can include water molecules, protonatable amino acid side chains, and peptide backbone δ^−^ groups (e.g., carbonyl O and amide N atoms). A simple example comprising two waters and two aspartate carboxylates is depicted in Figure 4. It is important to realize that unlike proton diffusion in bulk solution or at a flat interface, proton diffusion along a wire is limited to the single fixed pathway. Thus, the two “turn” steps depicted in Figure 4 are required in order to reset the wire in preparation for the next proton input; as discussed above, these rotation steps, because they require the breaking of a number of H-bonds, are likely to be rate-limiting. Such proton wires have been found in peptide ion channels like gramicidin A (Wraight, 2006), soluble proteins such as bovine pancreatic trypsin inhibitor, green fluorescent protein, and carbonic anhydrase (Shinobu and Agmon, 2009) (Shinobu et al., 2010) (Shinobu and Agmon, 2015), as well as transmembrane proton pumps and sinks like bacteriorhodopsin, cytochrome c oxidase, and F_1_F_0_-ATP synthase (Wraight, 2006).

One last modification of the Grotthuss proton hopping scheme in Figure 3 should be mentioned at this point in our discussion. The mechanism depicted suggests that the transition state involves the weakening/stretching of an O-H covalent bond (bond energy 110 kcal/mol) as the adjacent O:····H hydrogen-bond is strengthened/contracted. As suggested by its high conductivity, the activation energy for proton transfer in water is much lower than expected for such a process; in fact, it is only 2.7 kcal/mol (Cukierman, 2006). Agmon et al. have noted that this is equivalent to the strength of a single normal water-water H-bond (Lapid et al., 2005); in their most recent model for proton transfer in water (e.g., from left to right as in Figure 3), the transition state is reached by weakening three H-bonds on the right (Figure 1A, between the second and third hydration shell). Subsequent strengthening of an H-bond on the left yields the product proton, which has shifted to the right by one water molecule (Shevchuk et al., 2014). Stoyanov and Reed concluded that the proton’s excess positive charge is delocalized over both hydration shells in the H^+^(H_2_O)_6_ complex, depicted in Figure 1B (Reed, 2013); in this case, the stretching of H-bonds proposed by Agmon et al. would move the proton to the right by two water molecules.

It is interesting to compare proton mobility in water and in proteins. As mentioned above, long distance proton mobility in water (3-dimensional pathways) and along an interfacial surface (e.g., membrane, 2-dimensional pathways) can occur along a myriad of different pathways, whereas within a protein, protons move along single, stable proton wires. It is thus not surprising that the activation energy is much lower for the former (2–3 kcal/mol (Agmon, 1995)) compared to the latter (15–25 kcal/mol (Wraight, 2006)). This stems from the difference in transition states for the two processes: partial weakening/stretching of just a few H-bonds for the former vs. complete severing of several H-bonds during the two “turn” steps in the latter (Figure 4).

Additionally, it is interesting to compare the diffusion constants in bulk phases, cytoplasm, along the membrane surface, and within proton channels for water, H^+^, and other cations. We see from Table 3 that whereas Cs^+^_(aq)_ and water itself have *D* ≈ 0.2 Å^2^/ps, H^+^_(aq)_ diffuses 4x faster than water, and 4.5x faster than Cs^+^_(aq)_. Similarly, 2-dimensional proton diffusion along the membrane surface is also fairly efficient, being only 38% slower than 3-dimensional diffusion in bulk water. On the other hand, proton diffusion in the cytoplasm is impeded; while its maximum value is equivalent to that of water, it has been measured to be as low as 5x slower than water. Similarly, the slow diffusion of protons in reverse micelle nanodroplets was recently reported (Sofronov and Bakker, 2020). While water diffuses at least as fast in the cytoplasm as in the bulk phase, protons do not; it seems clear that Grotthuss water chains cannot form in the cytoplasm, either due to the crowding of macromolecules and cellular structural elements (Silverstein and Slade, 2019) or to the nature of water’s hydrogen-bonding network in nanoconfined spaces (Sofronov and Bakker, 2020). Finally, protons diffuse 15–20 times faster through the transmembrane gramicidin A channel than water or Cs^+^ do, and remarkably, protons diffuse almost as fast through the gramicidin A single proton wire as they do along the membrane surface. This shows that proton wires can be remarkably efficient in catalyzing proton flux.

**TABLE 3:** Diffusion coefficients (D) for cations and water, measured at 25°C (Wraight, 2006).

	D (Å^2^/ps)^a^		D (Å^2^/ps)^a^
H^+^ in bulk water	0.93	H^+^ in cytoplasm	0.04–0.22
H^+^ along membrane surface	0.58	H^+^ in gramicidin A^b^	0.3
Cs^+^ in bulk water	0.205	Cs^+^ in gramicidin A	0.015
H_2_O in bulk water	0.23	H_2_O in gramicidin A	0.02
H_2_O in cytoplasm	0.2–0.5		

a Equivalent to multiplying cm^2^/s by 10^4^.

b Gramicidin A is a naturally secreted pentadecapeptide antibiotic that folds into a β-helix and dimerizes in the membrane to form a cation channel.

Before we conclude our discussion of proton mobility, it is important to point out the predominantly ^6^ electrostatic nature of the hydrogen bonds that form water chains in the Grotthuss mechanism (Figure 3), and proton wires in proteins and peptides (Figure 4). One may view proton transfer as a series of protonation-deprotonation steps governed by acid-base equilibria (i.e., ∆pK_a_) that are specific to the polar covalent H-A bond. On the other hand, the H-bonds in water chains and proton wires are simple electrostatic attractions that can accommodate not just H^+^, but any ion of the proper size, charge, and ability to shed its hydration shell. In fact, while bacteriorhodopsin is a light-driven proton pump (Wickstrand et al., 2019), some bacteriorhodopsins pump sodium cations (Jakdetchai et al., 2021), and others chloride anions (Schobert and Lanyi, 1982). Similarly, while most F-type and V-type ATPases pump protons, some pump sodium cations (Mulkidjanian et al., 2008). Readers who are interested in more details on proton pumping by bacteriorhodopsin and mitochondrial Complexes I, III, and IV, and proton flow-driven F_1_F_0_ ATP synthesis, are referred to the following reviews: (Lanyi, 2004) (Wirth et al., 2016) (Sazanov, 2015) (Hirst, 2013) (Crofts, 2004) (Crofts et al., 2006) (Wikström and Sharma, 2018) (Brzezinski and Gennis, 2008) (Junge and Nelson, 2015) (Kühlbrandt and Davies, 2016) (Nath, 2006).

## 3 Discussion 2: Key Biochemical Reactions Influenced by pH and H^+^

### 3.1 Protein Side Chain Protonation, Salt Bridge Formation, and Ligand Binding

Now that we have surveyed the structure of the aqueous proton and its mobility both in solution and in proteins, we will explore the effects of H^+^ and pH on protein structure and function. A key feature that stabilizes protein structure is the salt bridges that form due to the electrostatic attraction between protonated cationic amino acid side chains (his, lys, arg, and N-terminal) and deprotonated anionic side chains (asp, glu, cys, and C-terminal). Of course, in order to form salt bridges, the pH must be high enough to deprotonate the −COOH or −SH group, but still low enough to keep the cationic side chains protonated. As we shall see, such salt bridges figure prominently in driving ligand binding (e.g., hemoglobin + O_2_, histone + DNA) and enzyme catalysis (e.g., chymotrypsin).

The classic example of the influence of pH on protein function is hemoglobin’s Bohr effect (Bohr, 1904) ^7^(Adair, 1925); for an excellent modern review, see (Eaton et al., 1999). This effect is described in detail in just about every biochemistry textbook published since the mid-20th century. Here we will cover only the basic idea, namely that as pH goes down, hemoglobin’s oxygen binding equilibrium constant goes down; in other words, H^+^ inhibits oxygen binding. This physiologically important effect allows hemoglobin to release more oxygen to tissues that function under low-oxygen or high CO_2_ conditions, both of which are characterized by lowered blood pH.

The structural underpinning of the Bohr effect lies in a number of salt bridges ^8^ that exist in hemoglobin’s deoxy conformation, but not when oxygen is bound; the conformational change that occurs upon oxygen binding moves the participating side chain ion pairs too far apart (Perutz, 1970). The existence of these “extra” salt bridges stabilizes the deoxy form relative to the oxy form, thus lowering myoglobin’s O_2_-binding equilibrium constant and enhancing oxygen release. The cationic member of all of these Bohr salt bridges is always histidine, featuring p*K*_a_ values between 6.5 and 7.9 (Sun et al., 1997). Hence, as pH is lowered from 8 to 6, these histidines become more protonated and more likely to form extra salt bridges in deoxy-hemoglobin; this extra stabilization of the deoxy form accounts for hemoglobin’s decline in oxygen affinity at lower pH.

Histone-DNA binding is another important example of the influence of protonation-dependent salt bridge formation (Isenberg, 1979). Histones are highly enriched in cationic lysine and arginine residues, allowing them to bind polyanionic DNA with high affinity (Record et al., 1978) (Fenley et al., 2010). Histones act as spools around which DNA winds to create nucleosomes, which in turn are organized into tightly packed chromatin in the nucleus. This packing of DNA protects the polymer from physical and chemical damage; on the other hand, for a gene to be transcribed or replicated, DNA must be unwound and released from histone. One way to trigger this release would be to deprotonate the cationic histone side chains, however, because the p*K*_*a*_ values of lysine and arginine side chains are above 10 (Table 2), this will not happen in the nucleus (pH 7.3, Table 1). Instead, to unwind DNA and activate transcription or replication, histone lysine side chains are neutralized (+1 to 0) by acetylation or methylation: lys-NH_3_^+^ → lys-NH-CH_3_ or lys-NH-CO-CH_3_ (Shahbazian and Grunstein, 2007) (Fenley et al., 2010).

### 3.2 Impact of H^+^ on Biochemical Kinetics: Chymotrypsin

**FIGURE 5:**
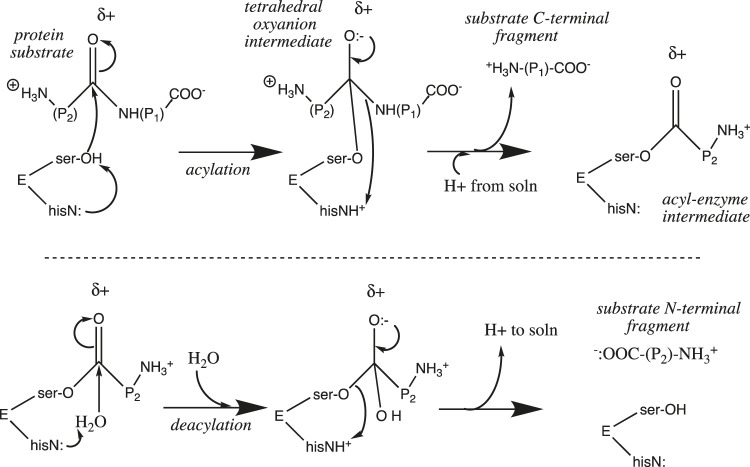
Chymotrypsin catalytic mechanism.

The rate of a reaction that features a proton at or before the rate-determining step (e.g., the reverse of Eq. 7) is pH-sensitive, as [H^+^] appears in the rate law. Similarly, the rate constant of an acid-catalyzed reaction increases with [H^+^], as the presence of a proton stabilizes the transition state and lowers its activation energy. For enzymes, the situation is a bit more complicated. Proton transfer is the most common enzyme-catalyzed reaction (Kirby, 1997) (Kraut et al., 2003), appearing in well over half of enzyme catalytic mechanisms (Jencks, 1987). Chymotrypsin is a “textbook” case ^9^ of an enzyme that utilizes general base catalysis as the key component of its catalytic mechanism. It is a serine protease that catalyzes the hydrolysis of amide and ester bonds. The active site catalytic triad features the serine_195_-OH nucleophile which is base-catalyzed by the adjacent histidine_57_-imidazole≡N, whose protonated state is stabilized by the adjacent aspartate_102_-COO:^−^. Once deprotonated by his_57_, the ser_195_-O:^−^ nucleophile attacks its substrate amide (or ester) carbonyl group (Figure 5). Upon collapse of the tetrahedral oxyanion intermediate, the RNH:^−^ leaving group is protonated by his_57_≡NH^+^ as well as a proton from solution as it leaves. A covalent acyl-serine enzyme intermediate is left behind at the active site. This is then hydrolyzed in an identical second series of steps featuring water as the base-catalyzed nucleophile and ser_195_-O:^−^ as the leaving group (Figure 5).

Proton transfer figures prominently in the catalytic mechanism at two points: the nucleophile is strengthened upon deprotonation by his_57_ ≡ N:, and loss of the leaving group is facilitated upon protonation by his_57_ ≡ NH^+^. In addition, Figure 5 does not show the substrate binding step that precedes catalysis. This is facilitated by an enzyme conformation that features a deep cleft leading from the external solution to the buried active site. Once the substrate diffuses into this cleft, it is bound by the enzyme with a precise orientation placing the ser_195_-OH near the substrate carbonyl carbon, and the δ+ amide protons near enough to electron-withdraw from the carbonyl oxygen and stabilize the tetrahedral oxyanion intermediate (Figure 5). This particular conformation is in turn stabilized by, among other things, a particular salt bridge between chymotrypsin’s N-terminal (ile_16_)-NH_3_^+^ and its asp_194_-COO:^−^. Above pH 8, the N-terminal is deprotonated, this key salt bridge is broken, and the enzyme conformation changes so as to disallow substrate binding (due to blockage at the mouth of the substrate binding cleft). Furthermore, below pH 7 the critical base catalyst his_57_ ≡ N: becomes protonated and can no longer deprotonate the ser_195_-OH nucleophile. These changes account for the pH dependence of chymotrypsin.

### 3.3 Michaelis-Menten Kinetics and the Enzyme Activity pH Profile

**FIGURE 6:**
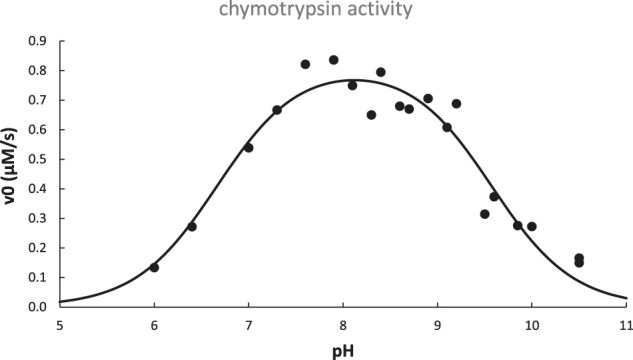
pH dependence of chymotrypsin-catalyzed hydrolysis of N-acetyl-L-tryptophanamide at 25°C. Data adapted from (Himoe et al., 1967) are fit to Eq. 13 with best-fit parameters: pK_a,lo_ = 6.67 ± 0.11, pK_a,hi_ = 9.57 ± 0.08, v_0,opt.pH_ = 0.82 ± 0.03 μM/s, *R* ^2^ = 0.93.

The activity pH profile of chymotrypsin is depicted in Figure 6; data are fit to the titration equation for a diprotic acid with two distinct p*K*_a_ values for H_2_A (p*K*_a,lo_) and HA^−^ (p*K*_a,hi_):

$$v_{0}=\;\frac{v_{0\left(opt.pH\right)}}{1\;+\;10^{\left(pKa,lo-pH\right)}\;+\;10^{\left(pH-pKa,hi\right)}}$$ (13)

As is typical for many enzymes, chymotrypsin’s pH profile is bell-shaped, with an optimal pH that is half-way between p*K*_a,lo_ and p*K*_a,hi_. From this we conclude that the monoacid is required for optimal enzyme activity, i.e., the acidic group responsible for p*K*_a,lo_ must be deprotonated, while the group responsible for p*K*_a,hi_ must remain protonated.

Enzyme pH dependence is often characterized using the Michaelis-Menten kinetic scheme, wherein non-covalent substrate binding is characterized by the Michaelis constant, *K*_m_, which for many enzymes ≈ the dissociation equilibrium constant of the non-covalent enzyme-substrate complex E·S, subsequent covalent bond changes in the catalytic step are characterized by the turnover number, *k*_cat_. A low value of *K*_m_ signifies tight substrate binding, while a high *k*_cat_ signifies rapid catalysis at the enzyme’s active site.

Referring again to chymotrypsin’s pH profile (Figure 6), it was later determined that p*K*_a,lo_ was due solely to the increase of *k*_cat_ with pH, whereas p*K*_a,hi_ was due to both an increase of *K*_m_ and a decrease of *k*_cat_ with pH (Kahana and Shalitin, 1974). Based on knowledge of the structure and catalytic mechanism of chymotrypsin, we can hypothesize that the active site his_57_≡NH^+^ titrates with p*K*_a,lo_ = 6.67, while its N-terminal ile_16_-NH_3_^+^ titrates with p*K*_a,hi_ = 9.57: his_57_≡NH^+^ must be *de*protonated in order to base-catalyze the ser_195_-OH nucleophile, while ile_16_-NH_3_^+^ must be *pro*tonated in order to form the key salt bridge that keeps the substrate binding cleft unblocked and stabilizes the optimal position of asp_194_, ser_195_, and other important nearby residues at the active site.

As shown in Figure 5, chymotrypsin’s acylation step requires proton influx into the active site, whereas the deacylation step requires proton efflux from the active site into the bulk solution. Because the substrate binding cleft is fairly large (after all, it must accommodate a protein substrate), proton mobility between the external bulk solution and the internal active site is not impeded; it can proceed via the Grotthuss water chains that are known to exist in bulk water. However, this is not the case for many other enzymes. The substrate for carbonic anhydrase, CO_2_, is small, and diffuses easily through the folded protein into the buried active site. On the other hand, the proton that is produced by the catalyzed reaction (CO_2_ + H_2_O → HCO_3_^−^ + H^+^) must diffuse through the protein along a specific proton wire that includes the critical residue his_64_ (Tu et al., 1998) (Shinobu and Agmon, 2009). In addition, a proton wire connecting the external bulk phase to the buried fluorophore has been mapped in Green Fluorescent Protein (Shinobu and Agmon, 2009) (Shinobu et al., 2010) (Agmon, 2005).

### 3.4 Nonspontaneous Proton Transfer Reactions can Be Catalytically Useful

The catalytic mechanism of chymotrypsin features proton transfer from a very weak acid (ser-OH) to a weak base (his-imidazole). ∆p*K*_a_ for this reaction is -8 (= 7{his} – 15{ser}), so it is exceedingly nonspontaneous, with *K*_eq_ = 10^−8^ and ∆G° = +11 kcal/mol at 25°C. Reactions between even weaker carbon acids and weaker carboxylate bases are not uncommon in some enzymes, giving ∆p*K*_a_ as low as −23! How could such incredibly nonspontaneous reactions be catalytically useful? As discussed recently (Silverstein, 2021), to get around this problem, enzymes employ several catalytic strategies that can be categorized as either ground state or transition state effects. The first ground state effect is the alteration of p*K*_a_ by local environment that we discussed above. For example, carbon acid substrates bound to the enzyme can have p*K*_a_ values 4–20 units lower than in aqueous solution; increases in base strength of 2–5 units are also found (Silverstein, 2021). Some of these nonspontaneous proton transfers can be brought to ∆G° ≈ 0 by this p*K*_a_ shift effect alone, but more generally, about 2/3 of the unfavorable free energy is supplied by this effect; this leaves about 4–14 kcal/mol to be supplied by other effects (Silverstein, 2021).

Enzymes have been found to use two transition state effects to lower the activation energy of highly nonspontaneous proton transfer steps. Menger proposed a *split-site* model of enzyme catalysis that allows calculation of the amount of spontaneous substrate binding free energy that can be applied to lowering the activation energy (Menger, 1993) (Menger, 2005). Readers are referred to these original papers for the details of this useful model, but suffice it to say here that this effect has been estimated to lower the activation energy by 6–18 kcal/mol (Silverstein, 2021). Additionally, Cleland proposed that normal ground state hydrogen bonds can become shorter, stronger *low-barrier hydrogen bonds* in the transition state (Cleland and Kreevoy, 1994) (Cleland et al., 1998) (Cleland, 2000). Such a low-barrier H-bond has in fact been confirmed in the histidine-serine active site pair in chymotrypsin and other serine proteases (Frey et al., 1994). Formation of such super-strong H-bonds could supply 7–25 kcal/mol toward lowering the activation energy of the proton transfer step (Silverstein, 2021).

Besides p*K*_a_ shifting due to local environment, the other ground state mechanism employed by enzymes is *effective molarity*, defined as the increase in spontaneity (or rate) when the proton donor and acceptor are optimized in space by attachment to a single molecule. Getting around the requirement for diffusion and collision in solution increases ∆*S* of the reaction, which can be envisioned as an increase in the “effective” molarity of the reactants. Using chymotrypsin as an example, although in aqueous solution imidazole would not be expected to deprotonate ethanol, the reaction becomes much more spontaneous by positioning the two molecules (histidine and serine side chains) optimally at the active site of the enzyme. In general, effective molarity can provide about 7 kcal/mol (range: 4–11 kcal/mol) toward lowering the activation energy of the enzymatic proton transfer step (Silverstein, 2021).

Summing up, of the 4–14 kcal/mol required to scale the activation barrier for nonspontaneous enzymatic proton transfer steps, at the lower end, any one of the three mechanisms outlined above (effective molarity, split-site substrate binding, low-barrier H-bonds) could drive the reaction forward. At the upper end, one or two of the three would certainly do the trick. Although it is beyond the scope of this survey, we should mention at least in passing that proton tunneling has also been shown to be important in some enzyme catalytic mechanisms; for recent reviews see (Klinman and Kohen, 2013) (Nagel and Klinman, 2006) (Hammes-Schiffer, 2006).

To close this section, there is an intriguing example of effective molarity that appears in some proton transfer enzymatic steps; it is sometimes referred to as the “swivel/flip” mechanism. The basic idea is depicted in the proton wire in Figure 4, showing how a carboxylate can pick up a proton on one side, then swivel 180° to deliver it on the other side. For example, at the active site of triose (and hexose) phosphate isomerase, the catalytic base glu_165_-COO: deprotonates C1 of the triose, then swivels to reprotonate the substrate at C2 (Knowles, 1991). Phospho-mutases use a version of this swivel/flip mechanism (Herzberg et al., 1996) in which an active site histidine-imidazole-phosphate donates a phosphate at the C2-OH of glycerate-3P_i_, and then swivels 180° to remove one from the C3-OP_i_; for glucose-1P_i_, phosphate addition is at C6 and removal from C1. Finally, aconitase has been shown to remove OH^−^ from the C3 of citrate to create a bound intermediate *cis*-aconitate, which is then swiveled 180° in order to add water at C2 (Beinert and Kennedy, 1993), giving the product *iso-*citrate. The swivel/flip mechanism in these enzymes elucidates the ability to move a proton (or phosphate or hydroxide) from one carbon to an adjacent one by simply swiveling a carboxylate or imidazole side chain.

### 3.5 Impact of H^+^ on Biochemical Thermodynamics

#### 3.5.1 Lactate Dehydrogenase

The spontaneity of reactions that feature H^+^ as either reactant or product is influenced by pH. The reaction catalyzed by lactate dehydrogenase is a good example:


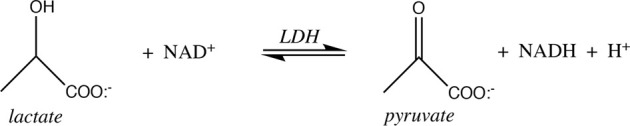


From the (chemical) standard reduction potentials of the pyruvate/lactate and NAD^+^/NADH redox couples (+0.224 V and −0.113 V, respectively), one can calculate a standard cell potential of −0.337 V, and ∆*G*° = −*nF∆E°* = +15.5 kcal/mol for lactate oxidation to pyruvate. Because this reaction must occur in aerobic tissues in order to metabolize the lactate created by fermentation in anaerobic tissues, it is clear that the standard free energy is not indicative of cellular conditions. Taking into account the difference in concentration of H^+^ going from the chemical standard state (1 M) to the biochemical standard state (10^−7^ M), lowers ∆*G°* by *RT*ln(10^−7^), or 9.55 kcal/mol, so ∆*G*°’ = +6.0 kcal/mol. This is better, of course, but still too nonspontaneous to be biochemically useful. The final adjustment to cellular conditions recognizes that lactate and NAD^+^ are generally present in excess over pyruvate and NADH. The exact ratios vary by tissue, oxygen concentration, and metabolic state, but Schwartz et al. reported the values in Table 4 above.

**TABLE 4:** Cellular concentrations of lactate, pyruvate, and ratios of [NAD^+^]_free_/[NADH]_free_, from (Schwartz et al., 1974).

	Avg. ± s.d	Range
[Lactate], µM	4,080 ± 2,600	1,400–10,400
[Pyruvate], µM	61 ± 43	20–168
[NAD^+^]_free_/[NADH]_free_	550 ± 300	240–1,300

Using the average values, we calculate that the biochemical reaction quotient (*Q’*) for lactate oxidation under cellular conditions is

$${\text{Q}}^{\prime }\;=\;\frac{\left[pyruvate\right]\left[NADH\right]}{\left[lactate\right]\left[NAD^{+}\right]}=\frac{61*1}{4080*550}=2.7\;\times \;10^{-5}\;$$ (15)

To calculate the effect of concentration on reaction free energy we use Eq. 16,

$$\Delta \text{G }=\;\Delta {\text{G°}}^{\prime }\;+\text{ RTln}\left({\text{Q}}^{\prime }\right)$$ (16)

Which at 25°C gives ∆*G* = 6.0 − 6.24 = -0.2 kcal/mol. Thus under average cellular conditions (Table 4), lactate oxidation runs very close to equilibrium (∆*G* ≈ 0), and slight changes in metabolic condition will alter the [pyruvate]/[lactate] and [NADH]/[NAD^+^] ratios so as to drive the reaction either forward or backward.

#### 3.5.2 ATP Hydrolysis

**FIGURE 7:**
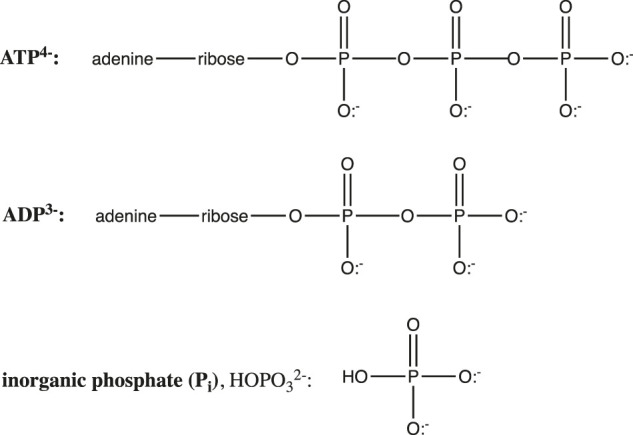
Structures of ATP, ADP, and inorganic phosphate.

The hydrolysis of adenosine triphosphate (ATP) is another crucially important biochemical reaction whose equilibrium is dramatically affected by pH. ATP is the “high energy” molecule whose hydrolysis supplies free energy to drive a myriad of nonspontaneous biochemical reactions, including muscle contraction, ion pumping, biochemical syntheses, etc. ATP hydrolysis yields the products ADP (Figure 7) and inorganic phosphate (P_i_, or HO-P_i_^2−^).

ATP and ADP are phosphoanhydrides (Figure 7), and the instability of these bonds, along with electrostatic repulsion within the polyanionic ATP and the extra resonance stabilization of the phosphate product are generally invoked to explain the spontaneity of the ATP hydrolysis reaction. In fact, as we shall see, the spontaneity of this crucial reaction is also controlled by ionic strength (*I*), Mg^2+^, pH, and the protonation state of ATP, ADP, and P_i_.

Added salt supplies cations that can screen the electrostatic repulsion within the ATP^4−^ (and ADP^3−^) polyanionic molecules. Thus, increasing ionic strength makes ATP hydrolysis less spontaneous, and it is important to take the typical cellular ionic strength of 0.2–0.25 M into account (Alberty and Goldberg, 1992) (Méndez, 2008) (Méndez and Cerdá, 2016). Also, Mg^2+^ binds with moderate affinity to both ATP^4-^ and ADP^3-^ (*K*_d_ = 0.12 and 1.1 mM, repectively, (Alberty and Goldberg, 1992)), serving to decrease ATP hydrolysis spontaneity in a similar fashion; consideration of a typical cellular Mg^2+^ concentration of 1–3 mM is thus also important.

But more important than ionic strength and magnesium is the influence of pH. It is clear from Figure 7 that in their fully protonated forms, ADP and P_i_ are triprotic acids, while ATP is a tetraprotic acid. p*K*_a_ values at 25°C and 0.2 M ionic strength are: 0.9, 1.4, 3.8, and 6.5 for H_4_ATP; 0.9, 3.8, and 6.3 for H_3_ADP; and 2.2, 6.65, and 12.4 for H_3_PO_4_ (Alberty and Goldberg, 1992). Thus, over the pH range 0–11, the protonation states of ATP, ADP, and P_i_ will change, giving different reactants and products for the ATP hydrolysis reaction, and thus different free energies:

$$\begin{array}{l} \mathrm{At pH 0}:{\text{H}}_{4}{\text{ATP }+\text{ H}}_{2}\text{O }\to {\text{ H}}_{3}{\text{ADP }+\text{ H}}_{3}{\text{PO}}_{4}\\ \mathrm{Around pH}3:\;{\text{H}}_{2}{\text{ATP}}^{2-}{\;+\text{ H}}_{2}\text{O }\to {\text{ H}}_{2}{\text{ADP}}^{-}{\;+\text{ H}}_{2}{\text{PO}}_{4}^{-}\\ \mathrm{Around pH}5:\;{\text{HATP}}^{3-}{\;+\text{ H}}_{2}\text{O }\to {\text{ HADP}}^{2-}{\;+\text{ H}}_{2}{\text{PO}}_{4}^{-}\\ \mathrm{Above pH}7:\;{\text{ATP}}^{4-}{\;+\text{ H}}_{2}\text{O }\to {\text{ ADP}}^{3-}{\;+\text{ HPO}}_{4}^{2-}{\;+\text{ H}}^{+} \end{array}$$ (17)

**FIGURE 8:**
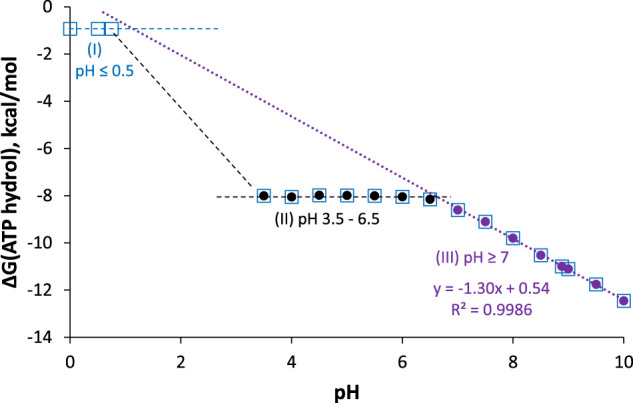
∆G for ATP hydrolysis as a function of pH; I = 0.25 M, [Mg^2+^] = 1 mM, T = 25°C. Data are taken from (Alberty and Goldberg, 1992) and (Méndez and Cerdá, 2016). Measured values are between pH 3.5–6.5 (black circles), 7–10 (purple circles), and pH 0 (open blue squares). Best-fit line for pH ≥ 7 points has intercept = 0.54 ± 0.17 kcal/mol, slope = −1.297 ± 0.020 kcal/mol/pH unit, *R* ^2^ = 0.9986.

As seen in the balanced equations in Eq. 17, ATP hydrolysis below pH 7 should be pH-independent, whereas above pH 7, ∆*G* should get more negative by *RT*ln(10) = 1.36 kcal/mol per pH unit increase. Furthermore, the products at pH 0 are all neutral and have no resonance forms, so this reaction should be considerably less spontaneous than at pH 2—6, where ATP is a polyanion with electrostatic repulsion, and ADP and P_i_ are stabilized by resonance. These predictions are all confirmed in the reported pH dependence of ATP hydrolysis free energy plotted in Figure 8.

The plot can be broken down into three pH regions: pH ≤ 0.5, where ∆*G* ≈ −1 kcal/mol; pH 3.5–6.5, where ∆*G* ≈ −8 kcal/mol; and pH ≥ 7, where ∆*G* gets more negative by 1.3 kcal/mol with each unit increase in pH (in agreement with the theoretical value of 1.36 kcal/mol/pH unit). Notably, at pH 7, ∆*G* = −8.6 kcal/mol (−36 kJ/mol), which is the biological standard free energy (pH 7, 1 mM Mg^2+^, *I* = 0.25 M) that should be used in biochemical thermodynamic calculations.^10^ It is also interesting to note that around pH 1.8 (between region 1 and 2), the predominant ATP hydrolysis reaction should be

$${\text{H}}_{2}{\text{ATP}}^{2-}{\;+\text{ H}}_{2}{\text{O }+\text{ H}}^{+}\;\to {\text{ H}}_{2}{\text{ADP}}^{-}{\;+\text{ H}}_{3}{\text{PO}}_{4}$$ (18)

Unlike region 3 (pH ≥ 7), ∆*G* for ATP hydrolysis around pH 1.8 should get more *positive* with increasing pH. No measurements have been reported (yet) in this region; it would be interesting to know if the predicted unusual pH-dependence is observed.

Hydrolysis free energy in these three pH regions shows that ATP is not a high energy molecule per se, nor can the weakness of the phosphoanhydride bond explain the spontaneity of ATP hydrolysis: Hydrolysis of fully protonated H_4_ATP (pH ≤ 0.5) is just barely spontaneous. On the other hand, hydrolysis of HATP^3−^ and H_2_ATP^2−^ (pH 3.5–6.5) is quite spontaneous, with ∆*G* ≈ −8 kcal/mol for both forms; this suggests that resonance stabilization of the H_2_PO_4_^−^ product is a key factor. Finally, the positive intercept of the fit-line for ∆*G* at pH ≥ 7 shows that if this particular reaction (i.e., ATP^4−^ hydrolyzed to ADP^3−^ and HOP_i_^2−^) *could* take place at pH 0, it would be nonspontaneous (∆*G* = +0.5 kcal/mol). This suggests that the hydrolysis of the ATP^4−^ tetra-anion, which is the reaction that occurs in most cells and organelles, is really only spontaneous due to the low concentration of H^+^! We have found no previous mention in the literature of this important conclusion.

#### 3.5.3 Chemiosmotic Theory and the Proton Electrochemical Gradient

One of the most important biochemical roles of the proton was proposed by Nobel Laureate Peter Mitchell (Mitchell, 1961) (Mitchell, 1966): In his Chemiosmotic Theory he posited that ATP synthesis is driven by the electrochemical proton gradient established across bioenergetic membranes, e.g., the bacterial plasma membrane, mitochondrial inner membrane, and chloroplast thylakoid membrane. The gradient is established by proton pumping that is driven by spontaneous electron transfer (oxidative phosphorylation in bacteria, mitochondria) or light (photophosphorylation in chloroplasts). All of these proton pumping complexes export protons from their topological inner (or N/negative) side to their outer (or P/positive) side (Azzone et al., 1993), leaving the inside phase negative and alkaline relative to the outside phase. Spontaneous proton influx through the F_1_F_0_ ATP synthase then drives ATP synthesis. Mitchell derived the thermodynamic equation showing that the energy stored in the proton gradient is a sum of its electrostatic component due to the transmembrane potential (∆ψ, negative inside) and its chemical component due to the concentration gradient (∆pH, alkaline inside):

$$\Delta {\text{µ}}_{\text{H}+}\;=\mathrm{zF}\Delta \psi +\mathrm{RTIn}\left(\frac{{\left[H^{+}\right]}_{in}}{{\left[H^{+}\right]}_{out}}\right)=\mathrm{zF}\Delta \psi \;-\;2\text{.}303\mathrm{RT}\Delta \text{pH}$$ (19)

where ∆µ_H+_ is the electrochemical potential for proton import, in kcal/mol (or kJ/mol), *z* = +1 (proton charge), *F* = Faraday constant, ∆ψ ≡ ψ_in_ - ψ_out_ is in volts, and ∆pH ≡ pH_in_ − pH_out_. Mitchell often expressed the driving force as a protonmotive force (pmf, in volts) by dividing both sides of Eq. 19 by *zF*:

$$\Delta {\text{µ}}_{\text{H}+}/\mathrm{zF}\;=\text{ pmf }=\;\Delta \Psi \;-\;\left(2\text{.}303\mathrm{RT}/\mathrm{zF}\right)\Delta \text{pH}$$ (20)

Using Eq. 19 we can calculate that for the influx of H^+^ across a bioenergetic membrane with ∆pH = 0.7 and ∆ψ = −120 mV, ∆µ_H+_ = −3.7 (2) kcal/mol H^+^ at 25°C. Thus, the import of 3 H^+^ would be sufficient to drive the synthesis of 1 ATP under biological standard conditions (∆*G°’* = +8.6 kcal/mol), and also under typical bioenergetic conditions (∆*G*_ATP synth._ ≈ 10.5 kcal/mol)^11^.

The actual H^+^/ATP coupling ratio is controlled by the subunit stoichiometry of the F_0_ proton channel portion of the F_1_F_0_ ATP synthase. Specifically, each F_0_ *c* subunit registers the flow of one proton as a torque-generating twist. Vertebrate mitochondrial F_0_ has 8 *c* subunits, hence the flow of 8 H^+^ causes a full rotation of 360°, which releases 3 ATP, one each from the three β subunits of the F_1_ portion of the synthase (Silverstein, 2014). This allows us to calculate that the thermodynamic efficiency of the mitochondrial ATP synthase can be, remarkably, ≈100%^12^ (= 10.5 × 3 ÷ (3.72 × 8))! Similar extremely high efficiencies have been calculated for bacteria (13/13.2 = 98%) and chloroplasts under certain conditions (13.2/15.6 = 85%) (Silverstein, 2014). Before we move on, it is worth reiterating that although Mitchell defined pH_in,out_ and *ψ*_in,out_ as pertaining to the bulk phases only, Williams hypothesized that the membrane surface pH was more important than the bulk pH (Williams, 1961) (Williams, 1978). Using bulk phase ∆pH and ∆*ψ* values have yielded thermodynamically impossible efficiencies of >100%, yielding theoretical support for surface-localized chemiosmotic theory^12^. As discussed above, there is also much experimental evidence supporting Williams’s surface-localized pH hypothesis.

F_0_ c-subunit stoichiometries have been found to range from 8 to 15 (Silverstein, 2014). For example, *n* = 10 for bacteria and yeast, and 14 for chloroplasts. It is interesting that vertebrate mitochondria and chloroplasts fall near the lower and upper extremes of c-subunit stoichiometries. Increasing c-subunit stoichiometry has the advantage of increasing the driving force for ATP synthesis (more protons flow per ATP), but at the same time, lowering thermodynamic efficiency. If chloroplasts, with their profligate *c*_14_ stoichiometry, evolved with abundant light to drive proton pumping, then thermodynamic efficiency may not have been an important evolutionary consideration in photosynthetic ATP synthesis (Silverstein, 2014).

#### 3.5.4 How Exactly Does Fermentation Acidify the Cell?

Chemiosmotic Theory explains how the maximum amount of ATP can be synthesized from the complete oxidation of sugar inputs under aerobic conditions. On the other hand, in the absence of oxygen as terminal electron acceptor, redox-driven proton pumps cannot function, so oxidative phosphorylation cannot occur. Under these anaerobic conditions, ATP is synthesized by substrate-level phosphorylation typical of fermentation. For a century or more, common wisdom has been that the acidification that occurs under anaerobic conditions, where pH can fall by 0.6 unit or more (Roberts et al., 1984) (Gout et al., 2001), is due to the organic acids produced by various fermentation processes (e.g., lactic, acetic, formic, succinic, butyric, and caproic acids). Although careful experimental results have repeatedly cast doubt on this conclusion (Gout et al., 2001) (Saint-Ges et al., 1991) (Menegus et al., 1991), it remains stubbornly common; it just makes too much sense that production of an acid should explain a decrease in pH. However, a careful examination of the complete balanced chemical equations for fermentative processes shows that protons are not in fact produced. For example, in lactate fermentation, glucose is split into two molecules of lactate, with production of 2 ATP:

$${\text{C}}_{6}{\text{H}}_{12}{\text{O}}_{6}\;\to {\;2\text{ C}}_{3}{\text{H}}_{5}{\text{O}}^{3-}{\;+\;2\text{ H}}^{+}$$ (21)

While it is true that lactate is produced along with protons, the 2 H^+^ products are consumed during production of 2 ATP at pH ≥ 7, as seen in Eq. 17. The balanced equation for the net reaction is then:

$${\text{C}}_{6}{\text{H}}_{12}{\text{O}}_{6}{\;+\;2\text{ ADP}}^{3-}{\;+\;2\text{ HOP}}_{\text{i}}^{2-}\;\to {\;2\text{ C}}_{3}{\text{H}}_{5}{\text{O}}_{3}^{-}{\;+\;2\text{ ATP}}^{4-}{\;+\;2\text{ H}}_{2}\text{O}$$ (22)

A similar balanced equation is obtained for the fermentation of ribose to lactate + acetate +2 ATP: there is no net production or consumption of H^+^. On the other hand, when the synthesized ATP is hydrolyzed at pH ≥ 7 to energize anabolic reactions, we see from Eq. 17 that H^+^ will be produced. So the acidification that occurs in lactate and acetate fermentation is due to ATP hydrolysis, and *not* to the production of the organic acids. Experimental evidence supporting this conclusion has been reported (Gout et al., 2001).

Along these lines, Raven and Smith proposed that the F_1_F_0_ ATP synthase evolved originally as an ATP-driven pump to extrude the protons produced during anaerobic fermentation (Raven and Smith, 1976). However, it was pointed out that the symporters that export the organic acid fermentation waste products from the cell co-export a proton for each acid molecule (Silverstein, 1989). Hence, there is no need for an ATP-driven pump to carry out a role that is already taken care of by the acid symporter. It is more likely that the original purpose of the F_1_F_0_ ATP synthase was to pump out protons that leaked into the primordial cells (Silverstein, 1989) from their acidic primordial environment (Woese and Fox, 1977) (Lake, 1988).

The other classic fermentation process converts glucose to two molecules each of ethanol and CO_2_ (and ATP):

$${\text{C}}_{6}{\text{H}}_{12}{\text{O}}_{6}\;\to {\;2\text{ C}}_{2}{\text{H}}_{5}{\text{OH }+\;2\text{ CO}}_{2}$$ (23)

While no protons are produced or consumed in Eq. 23, the synthesis of 2 ATP in this fermentative process will *consume* 2 H^+^ at pH ≥ 7, as shown in Eq. 17, but the subsequent hydrolysis of these 2 ATP to drive anabolic processes again leaves the proton concentration unchanged. So what then accounts for the acidification caused by ethanol fermentation? The answer, of course, is that CO_2_ is a weak acid: It reacts with water to produce carbonic acid, H_2_CO_3_. This in turn reminds us that it is not only anaerobic fermentation that can cause the pH to drop. Without the expulsion of CO_2_ by breathing, waste CO_2_, whether from aerobic *or* anaerobic metabolism would cause acidosis, and ultimately, death.

## Footnotes

1 The H^+^ ion is often referred to as a proton. Because the hydrogen atom has only one proton (no neutrons) and one electron, stripping off the electron to give H^+^ leaves only the proton.

2 The H+/3 ATP coupling ratio varies from 8—15, depending on species and organelle, as discussed below.

3 Regarding the important question of how equilibrium constants vary with size in nanoscale compartments (see (Goch and Bal, 2020) and references therein), Sawada et al. observed such a variation, but their nanoscale compartments were hydrophobic and substantially dehydrated (Sawada et al., 2009). Shon and Cohen did not observe this effect, except in their smallest, 250 nm radius compartment. (Shon and Cohen, 2012). Goch and Bal presented a detailed analysis of the conditions under which the variation is appreciable or insignificant, and used their model to explain the results of Shon and Cohen.

4 Note that the solvent actually changes the activity coefficients of the ionic products H^+^ and A^−^ (and to a lesser extent, the neutral reactant, HA); the activity-based *K*_a_ is solvent-independent. However, in organic chemistry and biochemistry literature, the molarity-based expression for *K*_a_ is almost always used (assuming all *γ* ≈ 1), so *K*_a_ is seen as being solvent-dependent.

5 Additionally, ∆*G*° for the acid-base reaction = -*RT*ln(*K*_eq_) = -2.3*RT*log(*K*_eq_) = -2.3*RT*∆p*K*_a_.

6 Quantum mechanical effects undoubtedly contribute some of the attractive force in hydrogen bonds, but these are often ignored, as they require solving appropriate Schrodinger equations. Models that include only electrostatic effects are generally quite successful in recapitulating physical systems. (Warshel, 1998), (Warshel, 1991).

7 For an English translation of Bohr’s article, see: http://www1.udel.edu/chem/white/C342/Bohr(1904).html.

8 The most well-characterized Bohr effect salt bridge is that between the β-subunit his_146_NH^+^ and the α-subunit asp_94_-COO: (Perutz, 1970), (Perutz et al., 1984), (Perutz et al., 1985).

9 Chymotrypsin is often the first enzyme mechanism covered in undergraduate biochemistry textbooks. See for example (Nelson et al., 2013).

10 As opposed to the values of -7.2 to -7.6 kcal/mol (-30 to -32 kJ/mol) that one often sees in the literature and textbooks.

11 Calculated using steady state mitochondrial matrix concentrations of 0.1 M P_i_ and [ATP]/[ADP] = 3, at 25°C (Silverstein, 2014).

12 Although reported values of ∆pH ≈ 1 are common in the older literature, recent measurements cluster around 0.1–0.4.(Chinopoulos et al., 2009), (Jaworska et al., 2021) Using ∆pH = 0.3 (instead of 0.7), the thermodynamic efficiency of proton-driven ATP synthesis in the mitochondrion would be 124%! This thermodynamically impossible value stems from the fact that the operative surface-to-surface ∆pH is undoubtedly much higher than the measured bulk-to-bulk ∆pH.
